# Bioactive Components, Applications, Extractions, and Health Benefits of Winery By-Products from a Circular Bioeconomy Perspective: A Review

**DOI:** 10.3390/antiox13010100

**Published:** 2024-01-14

**Authors:** Oana Emilia Constantin, Florina Stoica, Roxana Nicoleta Rațu, Nicoleta Stănciuc, Gabriela Elena Bahrim, Gabriela Râpeanu

**Affiliations:** 1 Faculty of Food Science and Engineering, Dunarea de Jos University of Galati, 111 Domnească Street, 800201 Galati, Romania; emilia.constantin@ugal.ro (O.E.C.); roxana.ratu@uaiasi.ro (R.N.R.); nicoleta.stanciuc@ugal.ro (N.S.); gabriela.bahrim@ugal.ro (G.E.B.); 2 Faculty of Agriculture, “Ion Ionescu de la Brad” University of Life Sciences, 3 Mihail Sadoveanu Alley, 700489 Iasi, Romania; florina.stoica@uaiasi.ro

**Keywords:** winery by-products, anthocyanins, antioxidants, value-added products, circular economy

## Abstract

Significant waste streams produced during winemaking include winery by-products such as pomace, skins, leaves, stems, lees, and seeds. These waste by-products were frequently disposed of in the past, causing resource waste and environmental issues. However, interest has risen in valorizing vineyard by-products to tap into their latent potential and turn them into high-value products. Wine industry by-products serve as a potential economic interest, given that they are typically significant natural bioactive sources that may exhibit significant biological properties related to human wellness and health. This review emphasizes the significance of winery by-product valorization as a sustainable management resource and waste management method. The novelty of this review lies in its comprehensive analysis of the potential of winery by-products as a source of bioactive compounds, extraction techniques, health benefits, and applications in various sectors. Chemical components in winery by-products include bioactive substances, antioxidants, dietary fibers, organic acids, and proteins, all of which have important industrial and therapeutic applications. The bioactives from winery by-products act as antioxidant, antidiabetic, and anticancer agents that have proven potential health-promoting effects. Wineries can switch from a linear waste management pattern to a more sustainable and practical method by adopting a circular bioeconomy strategy. Consequently, the recovery of bioactive compounds that function as antioxidants and health-promoting agents could promote various industries concomitant within the circular economy.

## 1. Introduction

In recent years, novel legislation about recycling and sustainability exploitation of locally accessible, underutilized raw resources in numerous countries has drawn attention to the circular bioeconomy for manufacturing high-value products [1]. Likewise, producers are looking for low-cost, simple technologies to stabilize raw materials due to their interest in natural ingredients. In 2020, the world produced about 260 million hectolitres (mhL) of wine, resulting in 20 million tons of biological by-products. One of the most frequently produced alcoholic drinks globally is wine, and approximately 259.9 million hL was produced in 2022. In Europe, Italy (49.1 million hL), France (46.6 million hL), and Spain (40.7 million hL) collectively produce 53% of the wine produced globally, with a substantial rise over 2019 [2]. Grapes, as the raw material for wine production, are among the most significant fruits grown worldwide and a rich source of phytochemicals [3]. According to the Statistical Report on World Vitiviniculture, the production of grapes was predicted to have been around 77.8 mt in 2018. Furthermore, 57% of cultivated grapes were used for wine-making operations, 7% were converted into dried grapes, and 36% were destined to be table grapes. Additionally, 39% of the world’s total grape production is generated in Europe, 34% in Asia, and 18% in America [2]. Nearly half of the world’s vineyards are in Spain (14%), China (11%), France (10%), Italy (9%) and Turkey (7%), respectively [4].

About 9 million tons of waste are produced along the wine processing chain, and their disposal significantly negatively influences the environment. These wastes are organic and inorganic wastes, wastewater, and discharge of greenhouse gases in the atmosphere. After obtaining of the grape juice, the residual pomace and stems are either composted or disposed of outside, which causes several environmental problems such as soil erosion, water pollution, unpleasant aromas, and greenhouse gas emissions. Using technology that reduce water consumption and facilitate by-product recovery helps the winery industry in limiting environmental damage while reducing the amount of waste produced. Waste can be viewed as a limitless source of biofertilizers as well as a resource that industry can employ to produce heat and electricity [5]. Several studies on the development of strategies and techniques for the valorization and application of vineyard by-products have been increasing in recent years, stimulating the interest of food scientists and industries [6,7,8].

In Europe, the usage of winemaking by-products is a pressing topic. These products have long been underestimated because there are no substitute uses that offer financial advantages. Traditionally, some of the waste was utilized as animal feed or fertilizer [9]. The use of wine pomace in distilleries to make various products, including ethanol, anthocyanins, tartrate, and grape seed oil, has become a viable alternative [10,11].

Due to the fact that the majority of wine producers sell their products to the energy industry, compost, and dumping, the wine industry has never been targeted or seen adversely despite being a significant source of pollution and an environmental issue. Winemaking generates a lot of waste and by-products in a short amount of time, which is equivalent to 30% (*w*/*w*) of the initial grapes. These wastes and by-products include wastewater, grape pomace, grape seeds, stalks, and wine lees [12]. Due to organic substances, pH, salinity, and heavy metal concentration, these by-products are regarded as highly damaging and negatively affect the viability of the environment and the economy [13]. These by-products contain phenolic compounds (phenolic acids, anthocyanins, tannins, and resveratrol), vitamins, water, lipids, proteins, carbohydrates, minerals, and compounds including fiber. The unexplored by-products from the wine industry could be an excellent source to extract products with an industrial base. Recovering high-value compounds from grape pomace reduces pollution while additionally providing phenolics, which might be used for developing synthetic color replacements [14].

It is becoming increasingly necessary to change from conventional techniques to sustainable circular approaches to value energy and waste through ecologically friendly procedures [15]. The term “circular bioeconomy” refers to methods in which waste materials produced at various stages of a bioprocess are simultaneously used in a cycle to create additional products. Bioeconomy processes that use renewable biomass, particularly that produced from crops and agricultural and industrial wastes, as source material for fuel production and chemicals to replace fossil fuel-based fuels and chemicals have become more important [16,17].

**Figure 1:**
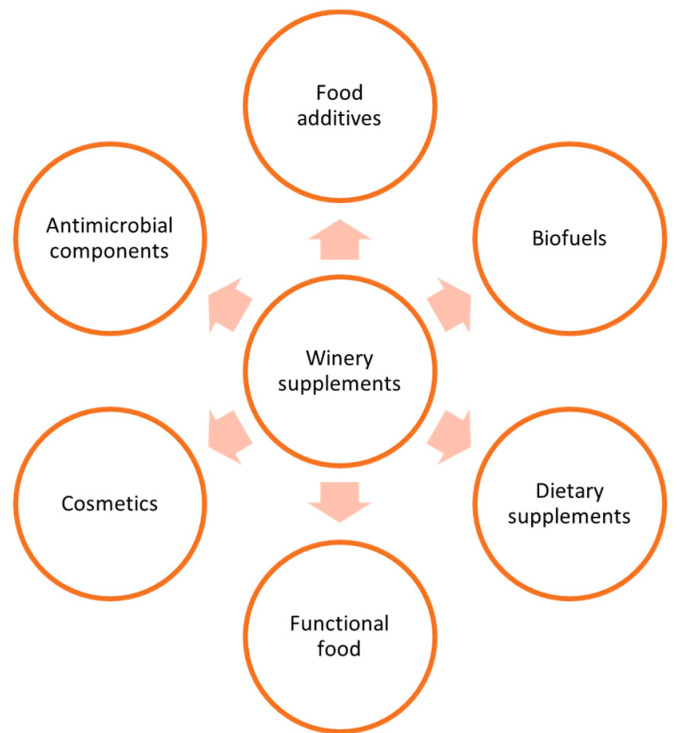
Utilization and importance of winery supplements.

According to studies, waste from wineries has a great potential to generate a variety of bioproducts. Various products, including antimicrobial compounds, food additives, functional foods, biofuels, dietary supplements, nutraceuticals, and cosmetics, can now be produced from winery waste thanks to industry diversification (Figure 1). Traditionally, the products were restricted to the fertilizer, dye, alcohol, and food industries. Waste generated by the wine industry may be evaluated for acceptable socioeconomic advantages linked with improved environmental conditions compared to the legislature practices now in use. The desire for green products, biofuels, bioethanol, and energy is particularly strong in regions with high energy costs. These costs may be significantly decreased by using winery waste as an energy source. This is because energy prices have increased significantly in recent years. The most effective strategy for addressing human health, environmental balance, climate change, and resource efficiency is integrated biorefinery. In order to use grape bioproducts in an integrated biorefinery without causing environmental harm, a successful economy and waste-to-energy views are required.

In the context of a circular bioeconomy, this article investigates the composition of winery waste, the extraction methods required, and the uses of such waste in the production of value-added products. The review aims to summarize and compile the most recent information regarding winery by-products as beneficial sources of bioactive compounds, the techniques used to extract these compounds, the potential health benefits of winery by-products, and the exploration of their numerous potential applications in different industrial sectors. Overall, this review provides a valuable contribution to the field of sustainable winemaking and circular economy.

## 2. Chemical Components in Winery By-Products

Grapes, one of the most widely cultivated crops, are primarily used to manufacture wine. A significant amount of solid organic waste, such as grape pomace, stems, and leaves, is produced throughout the winemaking process and must be appropriately treated and disposed of [18]. The chemical components of winery by-products are presented in Table 1.

**Figure 2:**
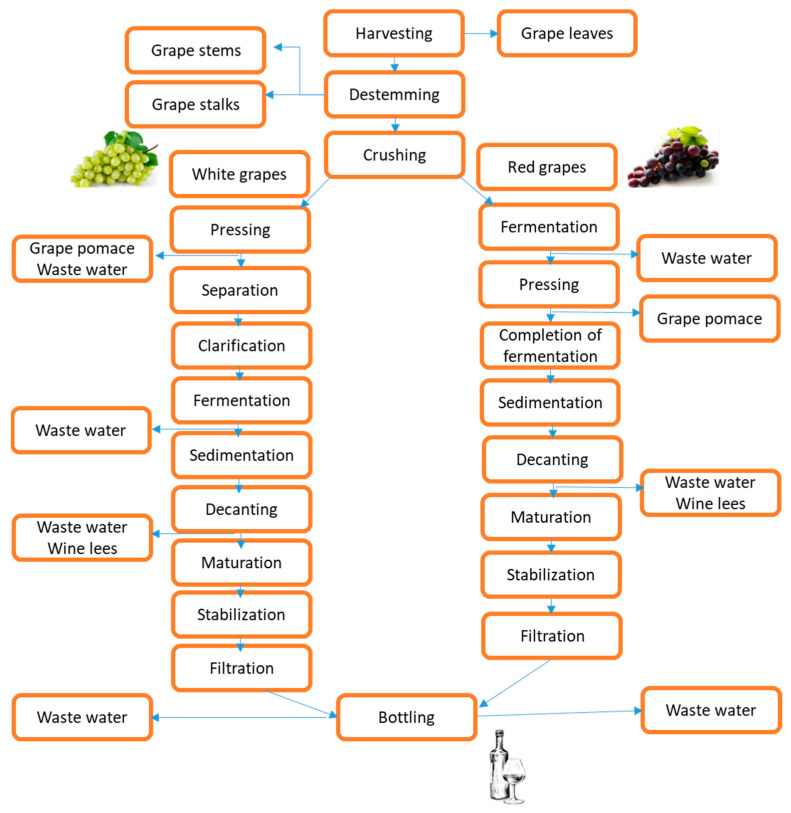
Schematic representation of winery by-product generation during wine production.

The grape berries are composed of between 85 and 92% pulp, 6 to 12% skin, and 2 to 5% seed [19]. Grape stems and pomace, composed of a combination of grape skin, seeds, and residual stem, are obtained after the destemming and crushing steps in the primary phase of winemaking, respectively [20]. According to Hogervorst et al. [21], grape stems comprise around 14% of the total solid waste, whereas grape pomace consists of up to 60% by weight and 20% to 25% of the grapes used in wine production. Stalks, pomace, seeds, and lees are solid by-products of industrial grape processing that may be of some commercial importance. However, they can be used as raw material for developing other commercially significant goods by utilizing extraction and purification procedures. The generation of various by-products at various stages of wine production is depicted in Figure 2. The increasing demand for organic vineyard waste and related products, which are already in the process of being developed, needs further investigation [22].

### 2.1. Grape Pomace (Marc)

In the early stages of grape juice, grape pomace is produced as solid waste. It comprises the grapes’ seeds, skin, and pulp, obtained through pressing and crushing them to extract the juice or must. One kilogram of grape marc is estimated to remain after 6 L of wine. Furthermore, 10% to 30% of the mass of crushed grapes comprises grape pomace. In addition to having high levels of alcohol and tartaric acid, grape pomace also has simple sugars, alcohol, polyphenols, tannins, pigments, and other economically significant compounds. Based on the grape variety and degree of maturity, the moisture content varies between 40 and 81% [23]. It is widely recognized that grape skins constitute the majority of grape marc, comprising 56% of the dry matter in red pomace and 28% in white pomace, respectively [24]. Gallates are found in small concentrations in the inner layer of grape skins compared to other phenolics in grape marc, such as anthocyanins and tannins. The grape skins include 5–12% structural proteins, 2–8% ash, 15% insoluble proanthocyanidins, 12% hemicelluloses and 20% cellulose, 20% acidic pectin components, 15% insoluble proanthocyanidins, and 5% substances soluble in dichloromethane [24]. However, the environment (temperature and soil), viticultural parameters (fertilization, maturity, grape variety, or harvest time), and winemaking technique all have an impact on the chemical composition of grape pomace [25]. Grape pomace mineral content includes iron, potassium, zinc, calcium, and phosphorus (1.8, 0.1, 0.1, 0.04, and 2.4%). The skin is also rich in minerals, with potassium (25.4% to 43.0% dry matter) being the most abundant mineral. Calcium (20.0% to 31.6% dry matter) and phosphorus (8.6% to 30.0% dry matter) are also present in grape skins. The vitamin C content of grape skin ranges from 0.5% to 1.2%, while grape pomace contains 2.6% dry matter [11].

The presence of cellulose, hemicellulose, lignin, and pectin (43–75%) in grape pomace makes it an essential source of fiber. It also contains considerable levels of protein, cellulose, and pectin components, as well as insoluble residues, according to research by González-Centeno et al. [26]. Peptic compounds comprise most of the polysaccharides in grape pomace cell walls, ranging from 37 to 54%. Another cell wall polymer that is prevalent in grape pomace and ranges in concentration from 27% to 37% is cellulose. Additionally, tocopherols, potent antioxidants, are present. On average, 65% of the entire grape pomace comprises grape skins. Grape skin has been established as a substantial resource of phenolic compounds; however, the exact yield is dependent upon the extraction method utilized and the vineyard practice. According to Ruberto et al. [27], grape skin waste extraction represents a novel class of valuable components that contains polyphenols and triterpenes and can be used to make nutraceuticals, medical treatments, and extracts for cosmetics. Traditional grape pomace distillation processes are used to create a variety of wine alcohols, distilled spirits, and liquors. In a fermentation process, the microorganisms can quickly utilize water-soluble carbohydrates. On the other hand, prior to undergoing enzymatic hydrolysis and saccharification, water-insoluble polysaccharides require an extra processing phase, which may involve pretreatment with acid or alkali or heating. Cost and energy are high for the pretreatment steps [28]. The constituents in grape pomace can be converted into chemicals, biofuels, and food ingredients. Pomace’s cellulose and hemicellulose content can be utilized for anaerobic digestion and fermentation to produce biofuels.

### 2.2. Grape Leaves

Grape stalks (leaves and shoots) are among the solid waste products produced by the wine industry. The primary by-product generated by the winemaking sector throughout the grape collection process is grape leaves. The leaves gathered from the industries are either burned or thrown at a landfill. Limited research has been conducted on the extraction and utilization of various types of sugar, phenolics, tannins, lipids, vitamins, flavonols, and organic acids; furthermore, no economically viable management approaches have been proposed [29]. These leaves are significant economically because they contain various organic acids and polyphenols. The food sector utilizes grape wine leaves, while the juice is recommended locally as an antiseptic eyewash.

### 2.3. Grape Stems

The grape cluster’s structure, which supports the fruit, forms the stem. Before the production process, the branch is removed to prevent any adverse effects on the flavor and aroma of the wine. Although grapevine stems are not used in vines, their production is closely related to wine, so they are regarded as an import winery waste with a high content of lignocellulosic material. Between 1.4% and 7.0% of the raw material handled are typically stems [30]. The grape stem is primarily utilized as animal feed and to increase soil fertility. Therefore, economically, it is not particularly significant. It was given less consideration than pomace and seed even though it is a potential waste for bioactive substances and is valuable for extracting essential substances [31]. There is no discernible difference between red and white grape varietals in terms of the moisture content of the stems, which ranges from 55 to 80%, depending on the variety. Proanthocyanidins, flavan-3-ols, hydroxycinnamic acids, stilbenes, monomeric, oligomeric flavonols, and other important compounds may be extracted from grape stems [32,33]. Regarding its potential application as a soil conditioner and fertilizer in agriculture, experts suggest the incorporation of winery waste.

### 2.4. Grape Seeds

The grape seeds, which account for 38–50% of the grape pomace on a dry matter basis and roughly 5% of the grape weight, are another significant by-product produced throughout the winemaking process. The seeds have 25 to 40% moisture, 36 to 46% sugars and polysaccharides, 2 to 7% organic acids, 13 to 20% oils and fatty acids, 4 to 6% phenolics (primarily tannins), and 6 to 6.5% nitrogen substrate. Dimeric, trimeric, and tetrameric procyanidins are also abundant in grape seeds, alongside monomeric phenolic compounds such as (-)-epicatechin, (+)-catechin, and (-)-epicatechin-3-O-gallate. Unsaturated fatty acids, such as linoleic acid (72–76%), are abundant in grape seed oil [34]. Vitamin E (tocopherol) is present in grape seeds at concentrations ranging from 10 to 20% dry matter, protecting polyunsaturated fatty acids and lipoproteins from lipid peroxidation. The nutraceutical qualities of grape seeds could make them valuable [35]. The winemaking industry releases nearly three megatons of grape seeds worldwide yearly [25]. A variety of bioactive substances that are directly linked to antioxidant properties can be extracted from seeds, including grapeseed oil, polyphenols, ethanol, methanol, and xanthan via fermentation, as well as the production of energy sources and natural antioxidants [36,37]. Recent studies suggest that grape seeds may be extracted to obtain hydroxybenzoic, cinnamic acid, and gallic acid derivatives, among other relevant compounds [38,39].

### 2.5. Wine Lees

Wine lees are produced at the end of the fermentation phase during the winemaking process. Wine lees are sediment-like materials, including active and dead yeast cells that gradually accumulate at the bottom of wine tanks following fermentation, storage, or authorized treatments. They also include the sediment that results from filtration or centrifugation of the product and makeup 2–6% of the final volume of the wine [40]. Polysaccharides, proteins, lipids, and other organic species with high oxygen demand (biologic oxygen demand, chemical oxygen demand) are in large quantities in wine lees. Wine lees are, therefore, regarded as an environmental pollutant. According to Alanon et al. [41], it belongs to the class of microbial biomass, which also includes a small amount of ethanol, organic acids (tartaric acid), phenolic compounds, inorganic materials, and microorganisms (mainly dead yeasts). Wine lees have been found to contain a significant amount of yeast. Enzymes that can speed up the hydrolysis and transformation of phenolics are secreted due to the high concentration of microorganisms, such as yeast. In addition, lees generate certain enzymes that catalyze the hydrolysis and transformation of polyphenolic substrates into highly valuable substances like gallic acid or ellagic acid [42]. Lees are crucial since they can interact with the polyphenol in wine, significantly impacting its color and organoleptic qualities. In terms of its chemical makeup, lees comprise phenolics (29.8 mg/g dry weight (DW)) and anthocyanins (6–11.7 mg/g DW) [41]. The first β-glucan isolation from wine lees using an environmentally friendly approach has been reported by Varelas et al. [22].

### 2.6. Wastewater

The wastewater is the vinasse generated in the distillery. Vinass, the liquid part of the wine lees, is made up of residual fermentation broth [43]. It has a low pH of 3.5 and roughly 58% water content by weight, making it a substantial source of polyphenol compounds [44]. It includes skin, seed, and dead yeast. Vinasse, a residue from the distillation unit, is directly correlated with alcohol production. A significant amount of wastewater emerges while cleaning and sterilizing the wine industry’s equipment. Compared to the vintage season, chemical oxygen demand and wastewater discharge are relatively lower during the non-vintage period. Wastewater from wineries is used to irrigate agricultural regions all over the world after receiving some basic treatment. The effects of treated and untreated wastewater pollutants on soil fertility and crop development are a major issue [45].

**Table 1:** Summary table of chemical components from winery by-products.

Winery By-Products	Chemical Components	References
Grape pomace	Protein Polymers (cellulose, hemicelluloses, lignin, pectin) Monosaccharides, Polysaccharides, Oligosaccharides Minerals (Fe, K, Zn, Ca, P) Tocopherols Polyphenols (anthocyanins, tannins, kaempferol, kaempferol-3-O-glucoside, quercetin 3-O-glucuronide, myricetin) Hydroxybenzoic acids (gallic acid) Hydroxycinnamic acids (caffeic acid, caftaric acid, p-coumaric acid, fertaric acid)	[11,25,26,27,46,47]
Grape leaves	Polyphenols (flavonols, tannins) Lipids Vitamins Organic acids	[29]
Grape seeds	Polysaccharides Hydroxybenzoic acids (syringic acid, vanillic acid p-hydroxybenzoic acid, protocatechuic acid) Polyphenols (anthocyanins, quercetin 3-O-glucuronide, myricetin, hydroxytyrosol) Stilbenes (glucosides piceid, resveratrol, astringin, viniferin) Vitamin E (tocopherol) Fatty acids Organic acids	[34,35,38,39,48,49]
Grape stems	Proteins Lipids Polymers (cellulose, hemicelluloses, lignin, lignocellulose) Polyphenols (flavan-3-ols, flavonols) Hydroxycinnamic acids Hydroxybenzoic acids (gallic acid, protocatechuic acid, vanillic acid) Stilbenes (glucosides piceid, resveratrol, astringin, viniferin)	[33]
Wine lees	Organic acids (tartaric acid) Anthocyanins Polymers (cellulose, hemicelluloses, lignin)	[41,42]
Wastewater	Polyphenol compounds	[50]

As a result, solid by-products from industrially processed grapes, such as grape pomace, leaves, stems, seeds, and wine lees, among others, are valuable economically. A more valuable by-product that represents the majority among the above presented is grape pomace. Because of its physical components as well as its alcoholic and tartaric richness, grape pomace is regarded as having significant economic value. Additionally, is abundant in phenolic acids, flavonoids, colorful anthocyanins, and tannins. Also, distilleries may use it as a substrate to produce tartrates and alcohol.

## 3. Innovative Applications of Winery By-Products

Utilizing waste produced by the wine industry to recover important phytochemicals is becoming increasingly popular, reducing waste from ending up in landfills and wastewater systems. Polyphenols, pigments, alcohols, unfermented sugars, tannins, and several other important substances can be found in considerable quantities in the waste produced by the wine industry. It fulfills the requirements to be considered a potential feedstock for the production of renewable energy sources due to its very high concentration of lignocellulosic components. Waste from the wine industry offers much potential for uses with added value through the extraction of valuable components through thermochemical and biological treatments as well as energy recovery. Utilizing this waste, distinctive products like biosurfactants—which may be utilized to clean up the environment—can be made.

### 3.1. Substrate Source for Microbial Fermentation to Produce Metabolites

Grape pomace can be fermented to produce cellulose, various hydrolytic enzymes, and exo-polygalacturonase (exo-PG). During the clearing processes, these enzymes are widely used in the food, paper, and pulp industries as well as in wine cellars [50]. *Phanerochaete chrysosporium* utilizes the lignin in the lignocellulosic waste from grape seeds and stems to produce laccase [51]. Salgado et al. [52] produced lipase, tannase, and protease, three industrially significant enzymes, using wastewater from wineries. To achieve the biodegradation of the combined wastewater from the olive oil mill and the winery, fungal species screening was carried out. The utilized *Aspergillus* sp. fungus culture displayed considerable removal of organic compounds from winery wastewater besides producing 1257, 284, and 3700 U/L of lipase, tannase, and protease, respectively.

*Aspergillus awamori* was used by Dáz et al. [53] to produce xylanase from grape pomace in submerged and solid-state fermentation, which increased the sugar contents but inhibited the production of the enzyme due to the high sugar concentration. By utilizing *Aspergillus awamori* in solid-state fermentation, grape pomace is a great source for producing hydrolytic enzymes such as pectinases, cellulases, and xylanases [54]. Moreover, grape pomace has the potential to serve as a substrate for the synthesis of microbial proteins. These may serve as an alternative source of high-quality protein for animal feed or as an alternative protein source for human nutrition, among other purposes [55].

As a potential substrate for lactic acid production, vinasse has been proposed, and a maximum concentration of 17.5 g/L has been determined [56]. Bustos et al. [57] discovered that *Lactobacillus pentosus* could continuously produce lactic acid from pre-treated vine shoot hydrolyzate. Yeast extract and corn steep liquor were included as extra vitamin supplements. *L. pentosus* transformed the glucose in the pretreated vine shoot hydrolysate into lactic acid. Interestingly, the same bacterial species fermented xylose into acetic and lactic acids. Adding additional nutrients, pretreatment vine shoot hydrolysate produced lactic acid at a productivity and yield of 3.10 g/L × h and 0.70 g/g of sugars, respectively. A final lactic acid concentration of 21.80 g/dm^3^ was produced by *L. pentosus* using a carbon substrate made up of vine shoot hydrolysate (18 g/dm^3^ xylose and 11 g/dm^3^ glucose) [58]. Investigation on wine lees might be an extra source of nutrients for fermentation were made. As a dietary supplement, red or white wines from the first or subsequent decanting phase were employed to stimulate lactic acid production when *Lactobacillus* spp. were present. For instance, wine lees (as a nutrient) from the second decanting produced 20 g/dm^3^ of lactic acid, while *Lactobacillus rhamnosus* produced 105.50 g/dm^3^ of lactic acid achieving a 2.47 g/dm^3^ h volumetric productivity [58].

Typically, wine lees are used to produce tartaric acid. Tartaric acid may be collected and provided to the pharmaceutical and food sectors by a variety of biological and chemical processes. According to Kontogiannopoulos et al. [44], wine lees residue from the wine industry can be processed by cation exchange resin to recover industrially significant tartaric acid while reducing the amount of unfavorable potassium content. About 74.9% of the tartaric acid was recovered, and the amount of water content, resin, and process pH significantly influenced this recovery. Using wine lees may obtain antioxidants, ethanol, and tartaric acid. The residual part which is abundant in yeast cells, might be converted into a feedstock for general fermentation.

According to Papadaki and Mantzouridou [59], citric acid can be made from solid waste from wineries. They combined grape pomace with olive processing wastewater, and *Aspergillus niger* produced citric acid on the enhanced substrate.

### 3.2. Source of the Phenolic Compounds

**Figure 3:**
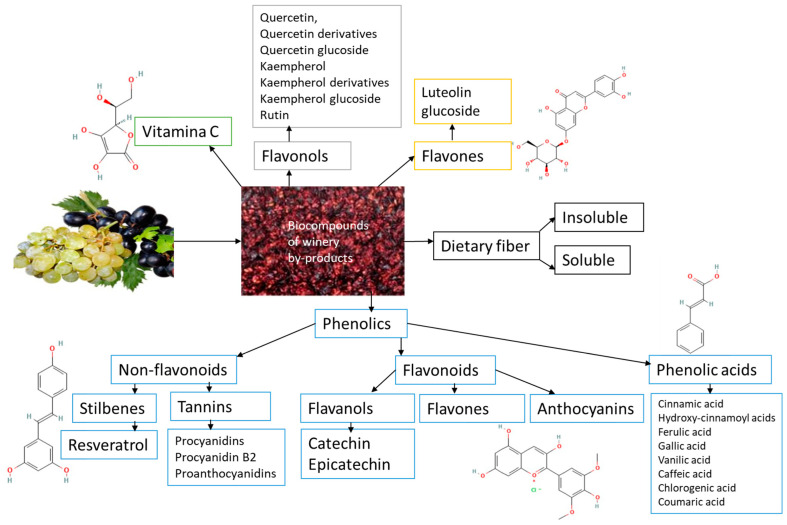
Scheme of selected winery by-products phytochemicals.

Significant amounts of residual phenols in raw plant materials from the wine and food sectors damage the environment. These substances produce a rise in biological and chemical oxygen demand in wastewater streams, rendering them unsuitable for irrigation since they impair germination qualities. Winery waste must be valorized because these phenolic compounds positively impact human health and are a potential source of enormous value. The largest class of phytochemicals, polyphenols, has been linked to numerous health advantages, including acting as potent antioxidants. Polyphenols are one of the most prevalent bioactive metabolites, accounting for about 70% of the bioactives. Their binding affinity, chemical structure, and quantity of aromatic rings classify them into two primary categories: flavonoids and non-flavonoids. The details of winery by-products phytochemicals are shown in Figure 3.

Among phenolic acids are found in grape residues, hydroxycinnamic acids the most abundant type found in grapes (fertaric, caftaric, and p-coumaric acids), and hydroxybenzoic acids (vanillic acid, p-hydroxybenzoic acid, protocatechuic acid, tannic acid, syringic acid, and gallic acid derivatives) [60]. Gallic acid, present in grape seeds, skin, and stems, is the most prevalent derivative of hydroxybenzoic acids and a precursor to hydrolyzable tannins [61].

Grape types, region of origin, and harvest time are some variables that affect the flavonoid concentration of residue used in the wine industry. Compared to white grapes, red grapes have a larger content of flavonols (kaempferol, quercetin). Similar flavonol profiles are found in the stems of white and red grape cultivars. However, the rest of the white-type plant material has very low levels of flavonols. Compared to individual wastes, the grape pomace (skin, pulp, and seeds) has a higher concentration of phenolics. Anthocyanins and phenolic acids can interact with flavonols to produce copigmentation. Quercetin is the predominant flavonol (0.92–5.66% dry weight), and gallic acid is the predominant phenolic acid (1.9–19.7% dry weight) in grape pomace [62].

The analysis of flavanol distribution across different grape varieties reveals that white and red grape winery residues contain the highest concentrations of catechin and its enantiomer epicatechin. The best source of catechins, among other winery by-products, is grape stems. Additionally, catechin derivatives such as epigallocatechin, gallocatechin, epigallocatechin gallate, and epicatechin gallate have been identified in grapes [63]. Most anthocyanidins have been identified from grape pomace and skin since they are primarily found in grape skin, where malvidin-3-O-glucoside and peonidin-3-O-glucoside are the most abundant compounds. Among anthocyanins, malvidin-3-O-glucoside is the most abundant, ranging from 10.40 to 16.50 mg/g DW [64]. Anthocyanins can react with flavonols either directly or via various aldehydes to produce more stable pigments. Parameters such as the type, quantity, pH, and degree of polymerization of anthocyanins influence color intensity [65]. Resveratrol is the most reported stilbene found in grapes and wine (3,5,4′-trihydroxystilbene)—stilbenes derived from trans-resveratrol (3,5,4′-trihydroxystilbene) that exist as glucosylated derivatives or oligomeric forms known as viniferins. Although their concentration decreases with ripening, stilbenes (piceid, pterostilbene, and piceatannol glucoside) are also present in grape leaves and skin. The polymeric substances resulting in anthocyanidins are referred to as condensed tannins, sometimes proanthocyanidins. These can be found in grape seeds’ skin, pulp, and solid portions. During crushing, maceration, and fermentation, these substances move into the grape pomace [63,66].

### 3.3. Natural Food Additives

Due to the multifunctional characteristics of winery biocompounds, they could be employed in food applications for producing new and valuable foods. Grape by-product extracts have functional properties that make them useful in various applications, including food products, the inhibition of pathogenic microorganisms, and the prevention of lipid oxidation. A potential benefit of dietary antioxidants derived from grape by-products is increased nutritional value of meat and bread goods, vegetable oil oxidative protection, and meat product color stability [67,68]. Grape pomace is a valuable waste traditionally used in Europe for oil extraction and a source of tannins and protein for animal feed. In meat products wine pomace extracts was added to enhance antioxidant, preservative and color-stabilizing properties [69]. Fractions of grape by-products have also been studied for usage in food and beverage products. The food sector may obtain natural antioxidants and additives, including tartaric acid, from wine-making resources. Smith and Hong-Shum [70] state that natural tartaric acid is used to improve biscuits, candies, jams, jellies, chewing gum, cocoa powder, and alcoholic drinks. Additionally, it has applications as an acidifier in the wine industry, as a preservative, and as an emulsifier in the bakery industry. Phenols derived from Merlot grape seed flour were utilized in the production of consumer-appealing pancakes, cereal bars, and noodles that exhibited potent antioxidant activity. It is essential to note that grape pomace is a significant source of fiber (43–75%), which consists of pectin, cellulose, hemicellulose, and lignin [25]. However, according to local regulations, only a few products containing grape pomace extracts are commercially available in various countries [69]. As a result of the integrated usage and low cost of marc processing, other products must be developed. According to Kalli et al. [68], dietary fiber was utilized as a supplement and may have prebiotic properties.

The impact of natural antioxidants derived from wine industry by-products has been investigated in a variety of raw materials for the food industry, including seaweed oil emulsion, sunflower oil, beef, pork, chicken, and turkey meat, and in ready-to-eat foods such as restructured meatballs, hamburgers, and sausages [71].

Marchiani et al. [72] assessed the application of grape pomace antioxidants in the cheese-making process before or after distillation. This research demonstrated the potential of grape pomace powder as a functional component and a novel way of producing functional cheese. Enocyanine called E163 in the food business, is another naturally occurring color from the grape skin anthocyanins. The European Food Safety Authority (EFSA) states that anthocyanins can now be used as food coloring in drinks, marmalades, candies, ice creams, and pharmaceutical items. Additionally, the potential of grape pomace as a dietary fiber source has been the subject of research as a potential functional ingredient in dairy products [73] as an alternative source of antioxidant dietary fiber to delay lipid oxidation in yogurt and salad dressings [74] and to decrease rancidity on ice storage in seafood [75], and as a potential fining agent for red wines to eliminate red wine tannins [73].

The potential uses of winery by-products over the past few years are listed in Table 2. Using products made from wine pomace can have sensory effects in addition to the pleasant and desired effects (color, texture). According to Acun and Gül [76], cookies’ acceptability was increased when grape pomace flours (seedless wine pomace, wine pomace, and seed flours) were added at a rate of 5%. Furthermore, wine lees have been assessed as a potential substitute for the most often utilized preservatives in meat products. Alarcón et al. [77] assessed the effect of adding wine lees at 2.5% and 5% concentrations to deer burgers packed in a modified atmosphere at 4 °C. Due to increased phenolic content and antioxidant activity, the fortification acted against lipid and protein oxidation.

Additionally, including wine lees changed the foods’ sensorial properties and had an antibacterial impact against psycho-trophic aerobic bacteria. After sensory investigation, the hamburgers exhibited characteristics of wine and bakery notes, which were thought to be pleasant at low intensity. This was because of the growth of benzene compounds, esters, and acids that were previously found in wine lees.

The shelf life of lamb meat was extended by grape seed and vitamin E, which reduced lipid peroxidation and meat discoloration by around 20% after the seventh day of storage [78]. Phenols also reduced lipid peroxidation and meat browning by sequential radical quenching and hydrogen donation [67]. At dosages equivalent to 20–100 g/kg of phenolics, red and white grape pomace were added to apple and orange juice, and antibacterial activities against *Z. rouxii* and *Z. bailii* were observed [79]. In order to stop food from spoiling due to the proliferation of bacteria (such as *Lactobacillus* spp., *Streptococcus* spp., *Leuconostoc* spp., and *Aeromonas* spp.), researchers concentrate on naturally occurring antimicrobial components. The growth of LAB, *Pseudomonas* spp., and psychrotrophic populations in pig patties was specifically retarded by grape seed extracts [80]. It has been demonstrated that the use of grape pomace in food preparations facilitated the fermentation process of *L. acidophilus* and *S. thermophilus* by enhancing the synthesis of lactic acid and decreasing the duration of fermentation [81]. Polyphenols from grape seeds and skin extracts have been demonstrated to prevent the synthesis of acrylamide during the frying of potato chips and in a simulated physiological system [82].

In addition to its nutritional significance, grape pomace extract may be utilized as a functional component in foods and beverages to bring health advantages. Today, cheese can be made with grape pomace powder, increasing the amount of phenols and antioxidant activity. Parallel to this, cheese proteolysis appears unaffected by adding grape pomace powder [72]. Wine pomace power or extract is added to dairy products like yogurt and cheese to increase their mineral content and stop lipid oxidation [74]. Grape pomace solutions (1, 2, and 3%) were used to fortify the coagulated milk. As the concentration of grape pomace increased, so did the total dietary fiber. Rosales-Soto et al. [83] enhanced the antioxidant activity and consumer acceptance of cereal bars, pancakes, and noodles by the utilization of Merlot grape seed flour phenols. Another study emphasized that grape skin powders are a good source of antioxidant activity, total flavonoids, and total phenolic compounds that can be successfully transferred to Caciotta cheese technology [84]. According to Li et al. [85] and Wang et al. [86], grape seed extract can potentially reduce the amounts of nitrite in dry-cured sausages and the amounts of nitrosamines that are generated. It may also prevent the development of N-nitrosodimethylamine. Implementing innovative food technologies has helped to reduce grape by-products, lessen the negative effects of conventional processes, and make it easier to produce valuable natural products that assure food sustainability and satisfy market demand. Moreover, Anghel et al. [87], based on a kinetic model and the survival rate of the bacteria, examined the impacts of various drying techniques on Băbească neagră grape pomace purée inoculated with *Lactobacillus casei* ssp. paracasei to enhance the functional properties of the grape pomace.

**Table 2:** Summary table of potential food uses of different forms of winery waste.

Form of the Winery By-Product	Food Product	Functional/Technological Benefits	References
Grape seed flour	Wheat bread dough	Incorporating greater quantities of grape seed flour into dough reduces water absorption, which subsequently impacts the dough’s stability and rate of development. The decreasing number index exhibited a progressive decline as the addition level increased and the particle size decreased.	[88]
Grape skin	Butter biscuits	Enhanced the apparent and plastic dough viscosity of the butter biscuits. The modulus of instant springiness and the modulus of elasticity were both reduced.	[89]
Grape pomace	Crackers	The addition of 5, 10, and 15% grape pomace led to increased dietary fiber content, as per Regulation (EC) No. 1924/2006, suggesting a functional food.	[90]
Grape pomace from white grapes	Wheat biscuits	The dietary fiber contents of addition levels up to 10% were considerably higher than those of the control samples, and they were also distinguished by considerably greater antioxidant activities linked to their phenolic contents.	[91]
Grape skin and seed flour	Muffins	Fortification employing phenols and fibers. Improved physical and sensory qualities.	[66]
Grape skin	Cookie dough	Decreased dough consistency and stability. Increased water absorption. The volume and thickness of cookies decreased.	[92]
Grape skin	Pasta	Total polyphenols and antioxidant activities are increased Better sensory evaluation	[93]
Grape seed	Roast chicken	Reduction of microbial growth and oxidation. Physical/color properties	[94]
Red and white grape pomace extract	Chicken meatballs	Decreased TBARS values during storage and processing at −18 °C under vacuum	[95])
Freeze-drying wine lees	Hamburger	Increase in antioxidant and antimicrobial activity and phenolic compounds in burgers.	[77]
Grape pomace	Salmon burger	Storage stability and dietary fiber content enhancements. A diminution in sensory characteristics.	[96]
Grape pomace	Pork sausages	The addition of 0.5 and 1% grape pomace to the formulation resulted in a reduction in lipid oxidation and color lightening over a period of 10 days under refrigeration conditions.	[97]
Red and white grape skin antioxidant dietary fiber	Yogurt	Acidity, total phenolic content, and antioxidant activity are higher than in the control group, whereas pH, syneresis, and fat are lower. Lactic acid bacteria, phenolic content, and antioxidant activity were constant during the three-week storage period.	[72]
Red grape pomace antioxidant dietary fiber	Salad dressing and yogurt	Total dietary fiber, polyphenols, and radical scavenging activity increased. Peroxide results for yogurt and salad dressing have decreased. Yogurt’s lactic acid percentage and syneresis values were stable during storage for three weeks at 4 °C.	[74]
Grape seeds extract	Ice cream	Enhancement of phenols, improvement of sensory qualities	[79]
Microencapsulated anthocyanins from grape skins	Light-formulated mayonnaise	Compared to the control sample, the panelist gave the newly light mayonnaises enriched with 10% dry vesicles a favorable evaluation because of the ruby color provided by the anthocyanins.	[98]
White grape skin	Model fruit juice	Enhancement of antioxidant activity and color stability Probiotic strains *L. rhamnosus*, *B. lactis*, and *L. paracasei* maintained their stability during storage	[39]
Red Grape Skin Extract	White Beer	Increase in the level of bioactive compounds (total polyphenols, total flavonoid contents) and the antioxidant potential of beer samples.	[99]

### 3.4. Active Ingredients in Cosmetics and Pharmaceutical

Grape pomace and seeds have antibacterial and antifungal properties, making them valuable components for skin care products [100]. It should be noted that vineyard wastes may include pesticide residues and heavy metals besides these benefits. As a result, the cosmetics industry should remove them before formulating cosmetics [101]. According to a study by Sun et al. [102], administering grape seed polyphenolic extracts could help protect the retina from hyperglycemia-related harm, possibly by reducing oxidative stress-mediated damage and activating the Nrf2 pathway. Due to the high content of bioactives and polyphenols with antiaging, skin depigmenting, and photoprotective properties, grape pomace extracts can be used as essential ingredients in cosmetic product formulation (emulsion, cream, lotion, liquid gel-based serum, toothpaste). According to Serea et al. [103], red grape peel extracts show an inhibition effect in vitro against α-amylase, α-glucosidase, lipase, and lipoxygenase, indicating the potential use of red grape skin extract in modifying the activity of enzymes linked to metabolic syndrome.

Grape pomace microemulsions are a valuable source of topical anti-inflammatory, antioxidant (tocopherol), antiviral, antibacterial, antifungal medications, and various local anesthetics. Compared to cream, gel, or lotion, Kumar et al. [104] report higher drug absorption via the skin. Grapeseed oil is utilized in the cosmetic industry due to its regenerative and restructuring characteristics. It is very light, captivating the skin and leaving no oily residue. Its antioxidant properties are essential for delaying epidermis aging [105].

Grape skins and seeds, both dried and lyophilized, as well as unfermented/semi-fermented and fermented varieties, contain numerous nutraceutical compounds, mainly polyphenols. This is a prevalent ingredient in infusions. Infusion of grape by-products is for drying the wet material. As a result, these infusions have the potential to function as nutraceutical components that promote health [106].

### 3.5. Food Packaging

Packaging protects food from microbial contamination and other detrimental conditions, such as physical damage, moisture, temperature and light. It is viable to contribute to the overall shelf life of perishable foods and guarantee their quality control by using natural colorants or anthocyanins. Ferreira et al. [107] investigated the addition of grape pomace extracts comprising 0.15% aqueous extract (primarily polysaccharides), 0.35 to 0.75% grape seed oil, and 0.15 to 0.35% grape skin extract (wax) into chitosan films. Chitosan films containing the aqueous extract are highly hydrophilic and silky. In addition, these films exhibited improved antioxidative properties while retaining their solubility in water and mechanical strength. The incorporation of wax improved the films’ flexibility and reduced their rigidity while enhancing their antioxidant properties without altering their solubility. The results indicate that grape pomace extract films containing chitosan are an appropriate substitute for synthetic materials. These films could also act as carriers for bioactive compounds. In addition, they can improve the shelf life of food. Shahbazi [108] investigated the possible applications of grape seed extract (1% *w*/*v*) in chitosan and gelatin films, as such and in coupled with *Zataria multiflora* essential oil from *the Ziziphora clinopodioides* plant. Due to their high phenolic content, both films exhibited strong antibacterial and antioxidant properties. Biodegradable active packaging is important to the food sector because it offers a strong barrier against chemical and microbiological contamination.

### 3.6. Biofuels

Biofuels such as bioethanol, biodiesel, biobutanol, and biogas could be made from waste materials generated by the wine industry. It is possible to use grape pomace as a substrate for bioenergy generation. The utilization of vineyard wastes for thermal decomposition results from the growing interest in using biomass as an energy source. From an economic standpoint, it was shown that pyrolysis, which produces less residue, was more practical than combustion. Volumes of 150 kg of biochar and 140 kg of biofuel were made from one tonne of grape marc [109]. The humidity is a drawback of winemaking waste since it lowers energy productivity. In this regard, other procedures, such as hydrothermal carbonization, must be used, which demands softer operating conditions (180–250 °C and 20–40 pressure). The majority of final products consist of a liquid phase that contains dissolved organics, a solid phase that is abundant in carbon (hydrochar), and a small quantity of gases [110]. By treating grape pomace, higher energy output in hydrothermal carbonization was realized. A recent study found that treating grape marcs hydrothermally is an effective technique to obtain CO_2_-neutral solid fuels straight in the wineries. An essential step forward would be the efficient disposal of vineyard waste and the high-value use of carbons, turning an environmental problem into a benefit for the business [111]. Anaerobic digestion is a viable method for removing waste from wineries since it uses less energy and is more effective. The anaerobic co-digestion of vineyard residues was studied using activated sludge, producing 65% methane and a yield of 0.4 Nm^3^/kg chemical oxygen demand [112]. Anaerobic digestion made methane from grape pomace, pulp, and seeds. The substrates were pulverized to boost their maximal degradability by up to 22% [113].

Polysaccharides and lignin were recovered from the grape pomace using an efficient accelerated solvent extraction technique. Following the pretreatment of water-insoluble polysaccharides, the released carbohydrates made bioethanol [28]. By employing ensilage to create ethanol, Zheng et al. [114] aimed to increase the digestibility of grape pomace. A higher ethanol yield was produced when *Escherichia coli* KO11 was used to ferment diluted acid-pretreated grape pomace.

To produce methane, Achkar et al. [115] investigated the impact of pretreatment procedures on grape pomace. The production of methane, which may be used to produce energy, heat, and a variety of biofuels, was boosted by the combination of temperature conditions and alkali pretreatment. Da Ros et al. [116] used an anaerobic treatment on wine lees, grape marc, and grape stems to produce methane. The most amount of biogas was produced by the use of wine lees and grape marcs.

Since yeast is a crucial ingredient in bioethanol synthesis, yeast biomass and bioethanol generation are intrinsically linked concepts. Through fermentation, yeast is a microorganism that can turn carbohydrates into ethanol. This method creates bioethanol, a sustainable fuel that can be used instead of gasoline [117]. According to Stanley et al. [118], *S. cerevisiae* (RL-11) has demonstrated potential for producing fuel ethanol from renewable energy sources. The capacity of *S. cerevisiae* to tolerate a wide pH range and lower risk of contamination makes its usage in the industrial ethanol production process highly popular.

According to European Council Regulation [119], wine lees shall be transferred to distilleries to make ethanol. This alcohol can make spirit liquors because it is high in aromatic compounds from wine. Yeast biomass, tartrates, and polyphenols are among the high-value components that may be recovered from the distillation by-product, distilled lees, or vinasses. Vinasses, on the other hand, pose a potential ecological issue when disposed of because they are high in organic content and have a high oxygen requirement.

### 3.7. Biosurfactants

Biosurfactants are surfactants of microbial origin that are amphiphilic and can decrease solutions’ interfacial and surface tension, contributing to developing emulsions.

Globally, additional research is being conducted concerning “green processes” that use renewable resources to produce different fuels and chemicals, like biosurfactants. There have been some investigations on manufacturing biosurfactants from waste from the wine industry. According to Cortes-Camargo et al. [120], vine shoots could be used to make biosurfactants, but this required various combinations of fermentation media comprised of grape shoot hydrolysate were subjected to a sequential pretreatment involving a specific strain of *Bacillus tequilensis*. *Lactobacillus plantarum* and *L pentosus* were employed to produce biosurfactants from pretreated vine shoot hydrolysate [121]. The optimum carbon source for forming extracellular and cell-bond biosurfactants was hemicellulose, which could be found in the pretreatment vine shoot hydrolysate. Using *L plantarum* and *L pentosus* in independent and combined cultures demonstrated a notable generation of biosurfactants. Bustos et al. [57] evaluated the viability of a vinasses-based growth medium by using *L. pentosus* to ferment hemicellulosic hydrolysates to produce both lactic acid and biosurfactants. Furthermore, there were no variations in the quantity of lactic acid recovered between the tested media and the control, which was made using yeast extract and corn-steep liquor, with no differences in the amount of lactic acid recovered.

### 3.8. Other Winery Waste Applications

The biocompounds from waste from the wine industries are biodegradable substances that serve as a source of nutrients for microbial development during solid-state fermentation. This works well for processing and turning waste into substrates for growing plants, mushrooms, and animal feed additives. These procedures do not harm the environment and are environmentally friendly. In order to produce compost abundant in nutrients suitable for use as a substrate for plant development in the fields of gardening, horticulture, and agriculture, the organic matter must first undergo a biological decomposition process known as composting.

There are numerous ways to compost vineyard waste, and they all differ significantly depending on the substrate used and the proportions of the substrates used. Carmona et al. [122] easily composted grape wine pomace and grape stalk in a 1:1 ratio (*v*/*v*) over 20–24 weeks. Plants like the petunia and geranium showed considerable development when the compost was employed as a decorative plant-solubilized substrate. With a pH of 7.35, the compost appears to be slightly alkaline. Additionally, it revealed significant levels of calcium, potassium, and nitrogen.

Wine lees are a waste product of the wine-making process containing yeast cells that can produce compost or animal feed. Brewing and the production of biofuels all produce significant quantities of yeast biomass as a by-product. It has been demonstrated to have potential applications in several industries and is a rich source of protein, carbs, and other nutrients. Yeast biomass can be utilized as an agricultural fertilizer or a protein source for animal feed. Yeasts are beneficial for bioremediation because they can metabolize various organic substances and withstand various environmental conditions. The treatment of wastewater containing organic contaminants is one instance of bioremediation utilizing yeast [123]. Using yeast, organic wastewater molecules can be broken down into carbon dioxide and water. The procedure not only removes the impurities from water but also creates yeast biomass, a source of protein and other nutrients [124].

Winery waste can either be used directly or treated with solid-state fermentation to create protein-rich biomass that can be added to the diet of animals (such as pigs, cattle, lambs, etc.). In the diet of lambs, sundried grape pomace (12.2%) successfully replaced oat bran and wheat bran [125]. The quality of the meat was unaffected by this change in diet.

Due to their vast surface area and improved binding affinity, the high organic carbon content of the sludge from the wine production process may result in effective adsorbents for heavy metals. For instance, a green adsorbent made from grape marc was tested for removing lead (a heavy metal) from contaminated effluents. It was established that pH had a significant impact on adsorption. At pH = 5.5 and 22 °C, the maximum lead adsorption capabilities of Merlot grape marc and Sauvignon Blanc grape marc were approximately 40 mg/g and 64 mg/g, respectively. However, more research is required to test the success of heavy metal removal [126].

Within the context of a biorefinery, wine lees have the potential to provide poly-3-hydroxybutyrate, ethanol, tartaric acid, and antioxidants, with the residual yeast-rich fraction being converted into a generic fermentation feedstock. In order to substitute commercial yeast extracts and crude glycerol, this study used wine lees as a nutrient-rich supplement medium, as carbon sources. The production of poly-3-hydroxybutyrate was substantially influenced by the wine lees hydrolysates’ free amino nitrogen content [127].

## 4. Winery Waste Bioactive Compounds Extraction Techniques

The main and most significant phase in recovering, isolating, and identifying molecules of interest from raw materials is extraction. By-products from wineries are distinguished by their high phenolic content, which has antibacterial, antiviral, anti-inflammatory, and antioxidant qualities that are advantageous to human health. Due to the qualities above, grape pomace is widely used in the cosmetic, pharmaceutical and food industries. Traditional techniques, including maceration, hydrodistillation, solid-liquid extraction, etc., have been utilized for a long time. These procedures are time-consuming and demand a sizable volume of solvent, making them unsuitable for industrial use. Due to these shortcomings, there was a severe need to develop newer, non-conventional extraction methods. Modern procedures include pulsed electric fields, supercritical, microwave, or ultrasound-assisted extraction. Compared to traditional methods, these techniques have a quick extraction time, use less solvent, and are simple to recover from. A summary of extraction procedures has been presented in Table 3 regarding the valorization of winery wastes and by-products.

### 4.1. Solid-Liquid Extraction (SLE)

The most common technique for polyphenol extraction from grape by-products is SLE with mechanical mixing. The SLE is the treatment method for bioactive plant food ingredients that has received the most attention since it is an affordable and accessible means to recover the desired compounds from a solid matrix like grape by-products. Due to their polar nature, grape pomace is soluble in polar protic solvents like ethanol or methanol. The factors important to its performance, such as particle size, solvent type, fluctuation in concentration gradients, temperatures, solvent/sample ratio, diffusion coefficients, and extraction time, define the optimal extraction. According to Bucic’-Kojic et al. [128], the extraction of polyphenolics from grape seed was significantly influenced by both the extraction solvent and temperature, with a mixture of 50% aqueous ethanol and an average of 45.11% catechin being the most effective.

### 4.2. Pulsed Electric Fields (PEF)

A technology that shows promise for recovering valuable molecules from food by-products and waste is pulsed electric field treatment. A powerful electrical field is present, exposing the substance between two electrodes. PEF is a non-thermal food preservation method that uses high-voltage electric pulses for a few microseconds to treat liquid or semi-solid foods positioned between two electrodes [129]. The occurrence of pores due to the electrical field’s stress on the membrane increases cell permeability. Electric field strength, pulse time, and pulse number are variables to be considered. In skins and pulp, the energy input in PEF is reduced compared to conventional techniques (mechanical or enzymatic), ranging from 1 to 15 and 20 to 100 KJ/kg, making it an environmentally friendly process [130]. The food matrix and the PEF treatment methods can significantly impact the concentration of the recovered antioxidative chemicals. Barba et al. [131] observed the effects of pulsed electric fields on the recovery of biocompounds selectively from fermented grape marc with the higher recovery of anthocyanins revealed.

Compared to extractions assisted by ultrasounds, this method enhanced extraction yields by up to 22%. The method has mostly been applied to extracting polyphenols from grape waste. The concentration of anthocyanins in red grape waste increased by 60% when pre-treated with PEF for 1 min at 25 °C and subjected to conventional thermal extraction for 1 h at 70 °C. Compared to untreated samples, 10% more polyphenols were extracted from white grape skins after PEF treatment at 20 °C. While the industrialization equipment is developing and PEF does not apply to raw materials, it appears to be a promising method to utilize as a pre-treatment to extract polyphenols from by-products [132].

**Table 3:** Overview of extraction processes for winery waste.

Winery Waste	Conditions and Technique of Extraction	Compounds	Remarks	References
Red grape skins	SLE, Central Composite Design using ethanol (38.06–96.93% ethanol) acidified with citric acid (0.01 to 2.64%), at 13.06–71.9 °C for 11.36 to 78.6 min	Total anthocyanins, total polyphenols, and antioxidant activity	The optimized parameters were 0.85% citric acid concentration, 85% ethanol concentration, temperature 57.39 °C, extraction time 52.14 min. 25 mg cyanidin 3 glucoside/g 37.41 mg gallic acid/g 17.2 mM Trolox/g	[133]
Grape pomace, skin and seed	SLE, 70% ethanol at room temperature, 20 min at 3500 rpm	Total polyphenols	650 ± 32µg gallic acid/g 550 ± 23 µg gallic acid/g 480 ± 34 µg gallic acid/g	[134]
Grape pomace	PEF, 1.2 kV/cm, 18 kJ/kg	Polyphenols	Depending on the temperature utilized, higher extraction yields	[135]
Grape pomace	PEF, power supply 40 kV—10 kA, frequency 0.5 Hz, 0–564 kJ/kg of energy input	Anthocyanins	Compared to UAE, the anthocyanins are 22% greater	[131]
Vine shoots	PEF, 13.3 kV/cm, 0–1500 pulses, 50 °C, 50–762 kJ/kg/3 h diffusion	Polyphenols	Total polyphenols may have increased up to twofold above untreated samples. Resveratrol (0.032 mg/g), kaempferol (0.156 mg/g), and epicatechin (1.747 mg/g)	[136]
Pomace (seeds, stalks, and skin)	UAE, time of 2.5, 5, and 10 min, pulse treatment 5 s on/5 s off, temperature 25, 40 and 55 °C, ultrasound amplitude 20, 30 and 40%, water, 5 g/L	Anthocyanins	25% increase in total anthocyanins Optimum conditions were temperature of 55 °C, ultrasound amplitude of 40%, 6 min of treatment	[137]
Grape pomace	UAE, temperature of 17 ± 3 °C, liquid-solid ratio of 5:1 mL/g, water as solvent, power of 50–150 W; time of 5–25 min, frequency of 40–120 kHz	Polyphenols	Optimum conditions were power of 150 w, time of 25 min, frequency of 40 kHz 12% to 38% of phenolic compounds	[138]
Grape pomace	UAE, 5 g sample/100 mL solvent, power 200 W, 40:1 (solvent: solid), 1:1 (water: ethanol), 45 °C, 10 min extraction, and 30 min stirring	Polyphenols	Ultrasound as an independent technique reduced polyphenol yields, but when combined with shaking extractions, yields of 2079.33 mg/100 g were obtained.	[139]
Vine shoots	UAE, 24 kHz, 400 W, 50 °C, 3 h diffusion (1010–3428 kJ/kg)	Polyphenols	An increase in total polyphenol yields up to 45%. Epicatechin (0.671 mg/g), kaempferol (0.097 mg/g), and resveratrol (0.024 mg/g)	[136]
Grape skins	MAE, 100–540 W, 3–10 min, 0–50, solvent (0–50% ethanol in water)	Polyphenols	The best polyphenol extraction was achieved using 540 W for 3 min and 50% ethanol.	[140]
Grape skins	MAE, 2458 MHz, 1000 W/L, (8–92%) ethanol, 30 min	Total polyphenols	104 mg gallic acid/g	[141]
Grape skins	MAE, power of 100–500 W, solvent (50–80% methanol in water) time of 5 and 20 min, temperature of 50 and 100 °C	Anthocyanins	Compared to conventional extraction, the optimal extraction conditions (500 W, 100 °C, and 40% methanol in water extraction solvent) resulted in a 5 h to 5 min reduction in extraction time.	[142]
Grape pomace	MAE, power of 1000 W, time of 10 min, distilled water solution acidified with 2% (*m*/*v*) citric acid in a ratio of 1:3	Total polyphenols, total anthocyanins, antioxidant activity	6.68 ± 0.05 mg gallic acid/g 1.32 ± 0.03 mg malvidin-3,5-diglycoside/g 23.84 ± 0.57 μmol Trolox/g	[143]
Grape seeds	SFE, Box-Behnken design, 80–120 bar pressure, 4–6 kg/h CO_2_ flow rate, 10–20% (*w*/*w*) co-solvent percentage	Polyphenols Proanthocyanidin fractionation (FI and FII)	Optimized conditions were: pressure 80 bar, temperature 40 °C, flow rate 6 kg/h and 20% co-solvent. The total phenolic content of 7132 mg gallic acid/100 g. FI (>1000 mg catechin/100 g) and FII (>800 mg catechin/100 g)	[144]
Grape pomace, skin and seed	SFE, 70% ethanol, a flow rate of 2 mL/min, 60 °C, and 250 bar	Total polyphenols	570 ± 10 µg gallic acid/g 603 ± 14 µg gallic acid/g 336 ± 28 µg gallic acid/g	[134]
Grape pomace	PLE, 60–140 °C, ethanol/water (30:70; 70:30, *v*/*v*)	Total polyphenols	When a mixture of ethanol/water at a ratio of 70% was utilized at 140 °C, the yield of polyphenol was significantly increased for both wet (16.2 g gallic acid/100 g) and dry (7.28 g gallic acid/100 g) grape pomace extracts.	[136]
Grape pomace	PLE, ethanol and water mixtures (acidified or not) (50% *w*/*w*), pure ethanol and acidified water at 40–100 °C	Total anthocyanins, total phenolic compounds	The best PLE conditions for bioactives extraction (50% ethanol-water pH 2.0, 40 °C) resulted in 10.21 mg of malvidin-3-O-glucoside/g and 35.30 mg gallic acid/g	[145]

### 4.3. Ultrasound-Assisted Extraction (UAE)

Recently, UAE has been used increasingly to extract bioactive compounds due to the cavitation effect, which enhances heat and mass transfer through plant cell wall disintegration. Increased shear from violent bubble implosion leads to more cell damage, solvent penetration, and extraction rate [146]. The increase in UAE extraction can be linked to the synergistic influence of different factors that influence the process, such as temperature, time, solvent, pressure, frequency and amplitude, power, solid-to-liquid ratio, etc. Enhancing the quantity and quality of pigment extracted from by-products, achieving optimal extraction with reduced processing time, facilitating mass transfer, permitting the use of “green” solvents (e.g., edible oils, ionic liquids), requiring less solvent, and employing environmentally friendly technology are the primary advantages of employing UAE for pigment extraction [147]. Da Porto et al. [148] compared supercritical fluid and ultrasonic extracts. Ultrasound extraction was performed for 4, 7, and 10 min at 20 kHz and 80 W of power. Under the evaluated conditions, a higher polyphenol content was found for 4 min at an ultrasound power of 80 W. González-Centeno et al. [149] evaluated the effectiveness of UAE and a traditional technique in the recovery of phenolic compounds and antioxidants from grape pomace using water as extraction solvent used and mechanical agitation. It was discovered that the properties of the extracts derived through both techniques were comparable. At 50 °C, however, the UAE method exhibited an eight times increase than the mechanical stirring technique (200 rpm).

### 4.4. Microwave-Assisted Extraction (MAE)

Antioxidants are extracted and isolated using MAE as a sustainable technique. Electromagnetic energy between 300 MHz and 300 GHz is primarily converted to heat via ionic conduction and dipole rotation. The solid-solvent mixture is heated using microwave energy, and the required chemical substances are then separated from the solvent using the MAE process. The foundation of microwave heating is non-ionizing electromagnetic radiation. A high yield is guaranteed because when microwave radiation permeates a material, it interacts with the polar components and produces heat due to dipole rotation. Solvent, extraction time, microwave power, temperature, and solvent volume are variables to consider throughout the MAE process. Various research has looked into using MAE as a pre-treatment before using the traditional solid-liquid extraction method to extract bioactive chemicals from grape pomace. This pre-treatment was found to work around the barrier to industrial application. In industrial applications of polyphenol extraction, MAE enhances yield and selectivity when employed as a pre-treatment [150]. Rocha and Noroa [143] extracted phenolic compounds from grape pomace using UAE and MAE in an acidic aqueous solution containing 2% citric acid as a solvent. Findings provide information on the overall concentration of the phenolic compound and the antioxidant activity measured by ABTS (2,2′-azino-bis 3-ethylbenzothiazoline-6-sulfonic acid). Moreover, DPPH (2,2-diphenyl-1-picrylhydrazyl) increased with time for both extraction methods, indicating that MAE provided optimal extraction conditions.

### 4.5. Supercritical Fluid Extraction (SFE)

By operating fluids at temperatures and pressures beyond their critical points, SFE technology exploits the mass transfer mechanism. A substance with good solvating qualities above its critical temperature and pressure is called a supercritical fluid. Typically, supercritical CO_2_ is employed throughout the extraction process. This is due to its comparatively low temperature (31 °C) and pressure (73.8 bar), inertness, odorless, non-toxicity, affordability, lack of flammability, and ease of removal from the final form. This method has been considered sustainable because no solvents are used.

Choosing the appropriate supercritical fluid, preparing the sample, incorporating modifiers, and preparing extraction conditions are crucial aspects to consider throughout an SFE process [151]. The polarity of SFE could be changed by co-solvents (ethanol) to increase extraction yields. Multiple studies have utilized this technique to enhance bioactives extraction. Ghafoor et al. [152] stated that co-solvents are required to extract polyphenols from grape skins and that ethanol concentrations greater than 6% are required to enhance the SFE efficiency. CO_2_ temperature and pressure significantly affect the extraction of total polyphenols and anthocyanins (*p* < 0.05). A potential cause is that the solvating capacity of CO_2_ rises as pressures and temperatures rise. In addition, an increase in temperature could enhance polyphenol extraction by increasing the solubility and diffusion coefficient of the solute. However, the temperature cannot be elevated indefinitely because polyphenols degrade above 50 °C. Based on published research, grape bagasse that had undergone supercritical extraction contained increased phenolic compounds and antioxidants. To extract more oil from grape seeds, supercritical extraction has also been employed as a pre-treatment, with ethanol serving as a modifying solvent. Supercritical extraction has been used in industries to extract grape seed oil. Supercritical extraction has been successfully used to recover 86% of the products from grape pomace, making it practical economically [148].

### 4.6. Pressurized Liquid Extraction (PLE)

Accelerated solvent extraction, another name for pressurized liquid extraction, is an extraction method typically used at high pressures and temperatures (over the boiling points of the solvents). High temperatures significantly impact analyte solubility, diffusion, surface tension, and solvent viscosity. Additionally, it reduces solvent consumption and extraction time, speeding up mass transfer and extraction rates [145]. However beneficial use of elevated temperatures in PLE may be, it may also occasionally prove detrimental, mainly when dealing with thermolabile materials like anthocyanins that break down at relatively low temperatures. Thus, when optimizing the process, these aspects must be considered. The component type to be extracted, the type of solvent, the temperature range, and the continuous pressure applied are all elements that must be considered for extraction efficiency.

Studies show that PLE best extracts procyanidins from red grape pomace at temperatures greater than 800 °C and 6.8 MPa. Additional studies using PLE treatment have revealed increased polyphenols in winery by-products. Since it was determined that the effects of additional pressure were negligible, each PLE experiment normally uses a constant pressure dependent on the food product (between 4 and 20 MPa). Various designs are necessary based on whether the PLE operates in a continuous or static flow regime [145]. Temperatures for thermolabile and thermostable phenolic compounds would be 40–60 °C and 7–22 °C, using ethanol as the most common solvent.

## 5. Potential Health-Promoting Benefits of Winery By-Products

The winery by-products have potential health-promoting effects because they contain bioactive compounds that have been demonstrated to reduce the potential for several illnesses, including diabetes, cardiovascular disease, and cancer.

### 5.1. Antioxidant Potential

Antioxidant activity is the bioactivity of polyphenolic compounds from wine by-products that is most significant. The antioxidative capabilities, which include eliminating free radicals, inhibiting lipid oxidation, reducing peroxide formation, and others, have been the subject of numerous research [153]. The antioxidant effect of grape pomace was studied in human keratinocytes exposed to UV radiation that generated oxidative damage. The results indicated that the cells pre-treated with grape pomace displayed a notably reduced increase in reactive oxygen species (ROS), specifically associated with the apoptotic process and protein levels [154]. White grape pomace’s antioxidant activity was also investigated concerning H_2_O_2_-induced oxidative damage in human colonic epithelial cells, and it was discovered that the grape pomace had a significant role in lowering ROS levels [155]. Brenes et al. [156] looked at the effects of giving commercial grape seed phenolics to hens. They found that the antioxidant activity in grape seed phenolics meals and excreta had a higher capacity to scavenge free radicals when compared to the control diet. It is possible that grape seed phenolics could represent an additional supply of antioxidants for animal nutrition (equivalent to a vitamin E diet) in accordance with the enhanced oxidative stability of chicken breast meat resulting from grape seed diets.

### 5.2. Cardioprotective Effect

A comprehensive term encompassing illnesses that impact the heart or blood arteries is cardiovascular disease. Cardiovascular disorders are important in oxidative stress pathogenesis and are regarded as the main disease marker for therapeutic interventions. The potential lipid (cholesterol, very low-density lipoprotein, and triglycerides) decreasing ability of grape pomace had been demonstrated by in vivo assay on Wistar rats. De Oliveira et al. [157] suggest that grape pomace may be a less expensive alternative for treating coronary heart disease. The fresh and fermented pomace of the Fetească neagră red grape variety, which is rich in condensed tannins, anthocyanins, and polyphenols, had a comparable effect on isoprenaline-induced infarct-like lesions by reducing enzyme markers of the cardiovascular system. Furthermore, elevated levels of serum antioxidants and oxidative stress markers were observed, primarily malondialdehyde, were downregulated [158].

### 5.3. Anti-Cancer Effect

The polyphenol present in grape pomace has been identified to block metabolic pathways that include angiogenesis, invasion, and metastasis, as well as proteases and drug-metabolizing enzymes of phases I and II. They also modify cell-cycle checkpoints, apoptosis, and receptor-mediated activities [159]. Pérez-Ortiz et al. [160] demonstrated that grape pomace extract at concentrations ranging from 5 to 250 g/mL inhibits the proliferation of fibroblasts and colon cancer cell lines (Caco-2, HT-29). The study showed that grape seed extract exhibited anti-tumor potential through the elevation of Ptg2 in Caco-2 cells and the downregulation of Myc gene expression in HT-29. The non-anthocyanin component of grape seed extract has exhibited the potential to inhibit the growth of colorectal cancer cells. Apostolou et al. [147] demonstrated prevention from reactive oxygen species (ROS)-induced DNA damage and a suppressive impact on cervical and liver cancer cell growth using grape stem extracts. They investigated the extracts’ antioxidant potential, their ability to stop ROS-induced DNA damage, and how they may impede the proliferation of cervical and liver cancer cells at low concentrations. They conclude that grape stem extracts have similar activity to grape seed extracts. According to Hamza’s [161] research, grape seed extract may have anticancer effects by reducing inflammation, increasing apoptosis, and blocking cell growth in hepatocarcinoma. It has been discovered that grape seed extract possesses substantial anti-tumor properties against various cancers, including lung, breast, colon, leukemia, and prostate tumors [162].

### 5.4. Anti-Hyperlipidemic Effect

Scientific research has shown how grape seed extract may control postprandial hyperlipidemia. Triglyceride and cholesterol levels are significantly reduced in male Wistar rats who receive 5 mL of grape seed extract per kilogram of body weight. There are many mechanisms by which the grape seed extract exerts its anti-hyperlipidemic properties. According to Adisakwattana et al. [163], grape seed extract, for example, inhibits the digestion and absorption of lipids. Suppressing pancreatic lipase, cholesterol micellization, cholesterol esterase, and bile acid-binding ability can also explain its activity. Similar outcomes were reported by Ishimoto et al. [164] for male golden Syrian hamsters given 20 g/100 g grape pomace flour. The authors suggested that inhibition of the 3-HMG-CoA reductase enzyme and reduction in chylomicron plasma concentration might both result in reduced intestinal absorption of dietary fat. Due to its antioxidant properties, grape pomace can aid in the prevention and management of atherosclerosis by inhibiting LDL cholesterol oxidation. In this regard, the impact of red grape pomace on rats’ ischemic heart disease caused by atherosclerosis was studied. Red grape pomace intake raised HDL cholesterol levels, which had an anti-atherogenic impact and decreased the size and quantity of atherosclerotic lesions [165].

By observing the decrease in adiposity and the improvement in insulin signaling, Rodrguez Lanzi et al. [166] investigate the positive health implications of lyophilized whole grape pomace and grape pomace extracts added to the meal of rats with metabolic syndrome generated by a high-fat, high-fructose diet. Entire grape pomace was shown to be more beneficial than grape pomace extracts at reducing elevated systolic blood pressure and triglyceride levels in the blood, and this may be because entire grape pomace contains more dietary fiber.

### 5.5. Gut Health

Gut microbiota is still a good indicator of intestinal health. The effects of long-term supplementation with Syrah, Cabernet Sauvignon, and Marselan grape pomace extracts rich in phenolic compounds on rat intestinal microbiota have been studied. According to Chacar et al. [167], feeding at concentrations of 2.5 and 5 mg/kg/d has specifically increased the proliferation of gut bacteria. It has also been demonstrated that prebiotic substances, including fructans and grape skin polysaccharides, promote the proliferation of beneficial microorganisms such as bifidobacteria and lactic bacteria in the intestines of pigs [168].

### 5.6. Anti-Hyperglycemic Effect

An high quantity of sugar in the blood is indicative of a medical condition called hyperglycemia. Type 2 diabetes and this disease go together. The carbohydrate digestion and absorption enzyme intestinal α-glucosidase can be inhibited to cure it. The yeast cells were prevented from using the α-glucosidase enzyme by 63% and 43%, respectively, when exposed to red and white grape pomace extract at a concentration of 10 µg/mL. According to Hogan et al. [169], grape pomace extracts inhibit glucosidase, a vital enzyme for breaking oligosaccharides and subsequent glucose absorption, preventing postprandial hyperglycemia in diabetic mice. The outcomes indicate a potential role for grape pomace-produced bioactive compounds in managing diabetes. In earlier research, the same authors found that mice fed an obesity-induced diet and grape pomace supplements experienced anti-inflammatory effects, but no reduction in oxidative stress was seen. The increased expression of the glucose transporter protein 4 (GLUT4) and the peroxisome proliferator-activated receptor g (PPARg) in adipose tissue, as well as the modulation of adipogenesis and an increase in adipose glucose uptake, were described by Costabile et al. [170]. Insulin sensitivity was enhanced in the participants of this study after consuming a 250 mL beverage containing red grape pomace. It was a randomized, controlled human clinical investigation.

### 5.7. Antimicrobial Effect

The antimicrobial and antiplatelet properties of extracts derived from *Vitis vinifera* wine have been comprehensively documented in numerous in vitro studies. These studies have identified a variety of phenolic compounds present in the extracts, including epicatechin, trans-resveratrol, flavonols, and gallic acid, all of which contribute to their antimicrobial activity [66,171]. Several investigations have shown the antibacterial potential of winery by-product extracts. So, regarding the antibacterial activity of winemaking by-products, Gram-positive bacteria exhibited a broader inhibitory spectrum and/or lower minimum inhibitory concentrations than Gram-negative bacteria [172]. Several other researchers have examined red wine and grape seed extracts’ antibacterial effects on a specific form of dental plaque biofilm. *Actinomyces oris*, *F. nucleatum*, *Streptococcus oralis*, *S. mutans*, and *Veillonella dispar* comprise this biofilm model [173].

Red wine extract solutions enhanced with grape seed extracts demonstrated the highest antimicrobial activity among all the extracts studied. Grape seed extracts possess effective antimicrobial effects, ascribed to their substantial levels of flavonoids and their derivatives compared to other wine extracts. It is considered that these chemicals are primarily responsible for the extracts’ high antibacterial action [173,174].

## 6. Conclusions

Grapes are considered one of the most developed and economically valued crops due to their role in winery industries. Winery by-products acquired from winemaking is a predominant source bioactive substances that offer long-term health benefits, such as antihyperglycemic, anticancer, cardioprotective, and antihyperlipidemic effects. In order to maximize resource utilization, recover and regenerate products and components at the end of their useful lives, and keep resources in use for as long as possible, this study has reviewed the potential use of winery wastes, such as vine shoots, grape pomace, and wine lees, as raw materials for various applications. The by-products obtained during the winemaking process might make a wide range of products with added value, including industrial enzymes, natural food additives, organic acids, biofuels, biosurfactants, etc. To determine if these processes are commercially viable, it is necessary to scale up them and examine their life-cycle assessment and techno-economic feasibility.

A combined process involving chemical, biochemical, and thermal treatments may be necessary to maximize the value of this agricultural waste and produce a low-cost raw material. However, numerous studies have been conducted using grape pomace for various applications for which small- and medium-scale industrial producers could use it. Hence, the potential for recovering bioactive components, including oil, from grape pomace provides a promising prospect for waste use from an ecological and economic standpoint. The food sector became more interested in grape pomace valorization, with pectin extraction as a strong justification for more in-depth research. Additionally, it was discovered that the two main components of grape pomace, dietary fiber and polyphenols, were helpful in food fortification. More investigation into wine lees as a source of valuable compounds might lead to better utilization of this by-product, supporting the circular economy perspective in the winemaking industry. These by-products still need to be processed in a food-grade, economical, sustainable way and maintain the desired functionality. This area must still be improved to make these by-products commercially viable. Wine lees’ high polyphenol content and the potential for adsorbing pesticide residues are two factors that contribute to the paucity of efforts made to valorize them.

Technologies that can efficiently handle the trash generated during the winemaking process have been developed with the production of value-added products from waste from the winery sector in consideration. However, there are still issues with processes and economics that need to be researched to bring these technologies to market. The use of winery by-products or their active ingredients in combination with other phytochemicals to enhance food processing has not been extensively studied. The circular bioeconomy can be successfully implemented by turning biowaste into goods with additional value. Waste from the wine industries can be effectively utilized to create commercially significant products by using the circular bioeconomy principle. However, the procedures for pretreating winery waste are still being developed, and appropriate attention must be made at all scales, from the laboratory to the industrial. A thorough grasp of compositional analyses of various winery wastes is necessary for knowledge advancement. Investigating various variables that could lead to higher-profitable products being produced from winery waste is crucial. Developing novel studies for the complete valorization of vineyard waste may establish a future market. The valorization of winery by-products enables one to offer the by-products a second life while lowering production costs and residual quantities. In reality, waste prevention/minimization and by-product valorization are critical measures for the food sector’s efficient management system and sustainability in national legislation, international regulatory frameworks, and waste management directives.

As a result, the valorization of winery by-products offers an interesting path toward sustainable resource usage and waste management in the winemaking sector. Wineries may contribute to a more circular and environmentally responsible approach by turning waste into valuable resources while doing so, creating new revenue streams, and encouraging the development of eco-friendly products. To implement sustainable practices throughout the wine sector and beyond, this review promotes the integration of winery by-products valuation.

## Data Availability

Not applicable.
